# Oral Administration of Edible Snail Extract Powder Prevents UVB‐Induced Skin Damage

**DOI:** 10.1002/fsn3.71215

**Published:** 2025-11-13

**Authors:** Jamyeong Koo, Sungmin Cho, Wonchul Lim, Tae‐Gyu Lim

**Affiliations:** ^1^ Department of Food Science and Biotechnology Sejong University Seoul Republic of Korea; ^2^ Department of Food Science and Biotechnology, and Carbohydrate Bioproduct Research Center Sejong University Seoul Republic of Korea

**Keywords:** edible snail, hydration, skin, UVB, wrinkle

## Abstract

Edible snail extract powder, which is known to hydrate and help heal skin, was evaluated to see if it could protect against skin damage from UVB via oral administration in an SKH‐1 hairless mouse model. Over a period of 117 days, mice were exposed to UVB radiation and given edible snail extract powder to assess its impact on skin health. Toxicity tests confirmed its safety, with no significant changes in body weight, organ weights, or serum markers. Physiological analyses showed that edible snail extract powder significantly improved skin hydration, reduced trans epidermal water loss (TEWL), and inhibited wrinkle formation. Histological examination revealed increased collagen deposition and reduced stratum corneum thickness. At the molecular level, edible snail extract powder restored the expression of hyaluronic acid synthases (HAS1, HAS2, HAS3), collagen‐related genes (Col1a1, Col1a2), and Sod, while suppressing the levels of Mmp‐1, a matrix‐degrading enzyme elevated by UVB exposure. These findings suggest that oral administration of edible snail extract powder might enhance skin barrier function and combat photoaging through antioxidant, anti‐inflammatory, and ECM‐preserving mechanisms. This study highlights its potential as a functional food ingredient for skin health, warranting further investigation into its molecular pathways.

## Graphical abstract

Oral administration of edible snail extract powder (SEP) significantly protected against UVB‐induced skin damage in SKH‐1 hairless mice. SEP improved skin hydration, reduced wrinkle formation, and restored the expression of key genes related to hyaluronic acid synthesis, collagen production, and antioxidant defense. These findings suggest SEP may serve as a promising oral functional ingredient for preventing photoaging and enhancing skin health.

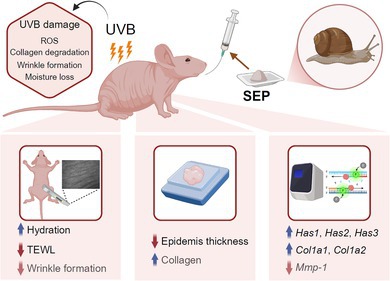

## 1 Introduction

The skin is one of the largest organs in the human body and is the most frequently exposed to ultraviolet (UV) radiation (Lee et al. 2024; Rabe et al. 2006). Exposure to UV light is known to induce a range of physiological changes in skin tissues (Kim et al. 2024; Naylor et al. 2011). Among these changes is photoaging, which refers to the premature aging of the skin caused by chronic UV exposure (Rabe et al. 2006). UV‐induced skin aging can lead to various symptoms, including increased trans‐epidermal water loss (TEWL), wrinkle formation, erythema, and pigmentation alterations (Naylor et al. 2011). Specifically, UVB radiation disrupts the stratum corneum, which is the outermost layer of the skin that helps keep water in, leading to more water loss and less moisture in that layer. This promotes epidermal dehydration and impairs skin cell function while also damaging and degrading structural proteins such as collagen, ultimately accelerating the formation of wrinkles (Fisher et al. 2002). Moreover, UV exposure reduces the levels of hyaluronic acid (HA) in the skin by enhancing the activity of hyaluronidases (HYALs), enzymes that break down HA (Dai et al. 2007). To alleviate UVB‐induced damage, sunscreens, antioxidants, and moisturizers have been widely used; however, these treatments are associated with issues such as allergic reactions (Addor et al. 2022). Therefore, this study aims to explore functional materials that offer sustained effects and ensure safety for long‐term oral administration.

HA is a high molecular weight polysaccharide that plays a crucial role in moisture retention and tissue protection within the skin and connective tissues. It is predominantly located in the dermis, where it helps maintain skin hydration and elasticity (Vasvani et al. 2020). HA is synthesized by hyaluronan synthases (HAS), of which there are three isoforms: HAS1, HAS2, and HAS3 (Nykopp et al. 2010). Among these, HAS2 is known to be the predominant one in dermal connective tissues, contributing significantly to HA synthesis and playing a critical role in maintaining skin hydration and structural integrity (Papakonstantinou et al. 2012). As a result, when the skin is exposed to UV light, it increases the activity of HYAL, which breaks down HA into smaller pieces, making it harder for the skin to hold onto moisture and speeding up water loss from the outer layer of the skin (Buhren et al. 2020). Additionally, UVB radiation induces the production of reactive oxygen species (ROS) and causes DNA damage in skin cells. This oxidative stress leads to inflammation and activates the mitogen‐activated protein kinase (MAPK) signaling pathway, which then boosts the activity of the transcription factor AP‐1 (Activator Protein‐1) (Heck et al. 2003) (Ujfaludi et al. 2018). AP‐1 activation increases the production of matrix metalloproteinases (MMPs), which are enzymes that degrade dermal collagen in the skin and other important structures (Jablonska‐Trypuc et al. 2016). The resulting loss of collagen leads to decreased skin elasticity and structural stability, further promoting wrinkle formation.

Snails produce mucus as an essential mechanism for survival. This mucus has been reported to possess beneficial properties related to wound healing and skin hydration (Tsoutsos et al. 2009). The secretion contains various bioactive components, including growth factors, antioxidants, and hyaluronic acid (Aouji et al. 2023). In addition, substances such as collagen, glycoproteins, and glycosaminoglycans have also been identified and are believed to contribute to their biological efficacy (Lim et al. 2024; Pfisterer et al. 2021). The SEP used in this study contains various amino acids, including lysine and glycine, which are essential for collagen synthesis. Lysine and glycine are known to contribute directly to collagen formation. Furthermore, previous studies have reported that treatment with snail mucin suppresses UVB‐induced ROS generation, suggesting that SEP may also contain diverse antioxidant compounds (Kim et al. 2022). As a result, Edible Snail Extract Powder (SEP) has recently gained increasing attention as a promising natural ingredient in the cosmetic industry. However, although increasing evidence suggests the effects of orally administered SEP, further clarification is still required. This study aims to explore how oral intake of SEP affects wrinkle formation from UV exposure, moisture retention, and skin elasticity in SKH‐1 hairless mice.

## 2 Materials and Methods

### 2.1 SEP Preparation

Edible snail extract powder (Lot. A2, 2024.07.27) was obtained from Age at Labs (Chungcheong‐do, Korea). SEP, which was derived from *Achatina fulica* (Bowdich, Achatinidae), was prepared through enzymatic hydrolysis, followed by enzyme inactivation, concentration, and spray‐drying. The preparation process was as follows: raw materials were thoroughly washed and visually inspected to eliminate foreign matter. Purified water was then added, and an initial extraction was carried out at temperatures above 100°C. After cooling the extract to 50°C ± 2°C, enzymatic hydrolysis was performed using food‐grade proteases and α‐amylase under continuous agitation for 10–12 h at pH 6.5 ± 0.5. The enzyme‐to‐substrate ratios were maintained within validated ranges of 0.4%–1.0% for proteases and 0.04%–0.1% for α‐amylase (w/w relative to raw material). Hydrolysis was stopped by heating at 95°C–100°C for 10–15 min to inactivate the enzymes. The resulting mixture was subsequently passed through fine filtration (≥ 300 mesh), concentrated, and spray‐dried when necessary to obtain the final powdered product.

### 2.2 Quantification of Lysine and Leucine via HPLC

For quantification of lysine and leucine, ~50 mg of dried sample underwent complete hydrolysis in 6 M HCl containing 1% phenol at 110°C for 24 h under nitrogen. After evaporation and neutralization, hydrolysates were derivatized with AQC (AccQ‐Tag chemistry) and analyzed by RP‐HPLC on a C18 column (4.6 × 150 mm). Separation was achieved with a linear gradient (aqueous buffer/acetonitrile), and detection was performed by fluorescence (Ex 250 nm/Em 395 nm). Calibration curves (≥ 6 points) were constructed using certified amino acid standards. The method was validated for linearity, precision, and recovery before analysis.

### 2.3 Animal Experiments

Animal experiments were conducted with the approval of the Animal Experimentation Committee of Sejong University (SJ‐20241016‐02). Six‐week‐old female SKH‐1 hairless mice (OREINET, Seongnam, Korea) were acclimated for 1 week prior to the experiment. The number of mice per group was set at 8, UVB irradiation was administered at an intensity of 50 mJ/cm^2^ in the first week, 100 mJ/cm^2^ in the second week, and 150 mJ/cm^2^ in the third week, followed by 200 mJ/cm^2^ for the remainder of the experiment. Starting on Day 1, SEP was given by mouth in amounts of 20, 40, and 80 mg/kg body weight (B.W.), dissolved in 0.9% NaCl solution. The selected concentrations were based on previous in vitro studies conducted in the laboratory, and as no safety issues were observed, the experiments were carried out using these concentrations. Both the untreated control group and the UVB‐only group received only 0.9% NaCl saline solution via oral administration. We collected dorsal skin, liver, spleen, and kidney after 117 days of administration.

### 2.4 Measurement of Skin Hydration and Trans‐Epidermal Water Loss

Skin hydration and trans‐epidermal water loss (TEWL) were measured during the experimental period to monitor changes in the dorsal skin condition of the mice. Measurements of skin hydration were taken at the early, mid, and final stages of the experiments, specifically on days 19, 73, and 117. We conducted eight consecutive measurements for each group using a Cutometer (Courage of Khazaka GmbH, Cologne, Germany). Trans‐epidermal water loss (TEWL) was also assessed at the same time points (days 19, 73, and 117) corresponding to the early, mid, and final stages of the experiment. TEWL was measured using a Tewameter (Courage and Khazaka GmbH, Cologne, Germany), with six repeated measurements per group.

### 2.5 Wrinkle Analysis Method

We used the Visioscan VC 20plus (Courage and Khazaka GmbH, Cologne, Germany) to capture wrinkle images. The mice were held in a fixed position during imaging, and measurements were taken at the same dorsal skin region for all mice. The values of skin wrinkles (SEw), skin smoothness (SEsm), skin scaliness (SEsc), and skin roughness (SEr) were quantified based on at least five repeated measurements.

### 2.6 Histological Analysis

To evaluate how the thickness of the epidermis and the amount of collagen in the skin changed, skin samples from the mice were preserved in 4% formaldehyde and placed in paraffin blocks. The paraffin‐embedded samples were sectioned at a thickness of 5 μm and mounted onto slides. The slides were colored with hematoxylin and eosin (H&E) and Masson's trichrome to measure the thickness of the epidermis and the amount of collagen in each group. Dorsal skin tissues were observed using a Leica THUNDER microscope at the Biopolymer Research Center for Advanced Material (BRCAN, Seoul, Republic of Korea).

### 2.7 Quantitative Real‐Time PCR Analysis

Total RNA from the dorsal skin of mice was extracted using TRIzol reagent (Thermo Fisher Scientific, Waltham, MA, USA). The extracted total RNA was then reversed transcribed into complementary DNA (cDNA) using the cDNA Synthesis Platinum Master Mix (GenDEPOT, Katy, TX, USA). Subsequently, quantitative real‐time PCR (qRT‐PCR) was performed. The thermal cycling conditions consisted of 40 cycles of denaturation at 95°C for 10–15 s and annealing at 55°C–60°C for 30–60 s. *Gapdh* was used as the reference gene, and its Ct values remained stable across all experimental conditions, validating its suitability as an internal control. The primer sequences used in this experiment are listed in Table 1.

**TABLE 1:** Primer sequences for qRT‐PCR.

Species	Gene symbols	Forward (5′ → 3′)	Reverse (5′ → 3′)
*Mus musculus*	*Gapdh*	CATCACTGCCACCCAGAAGACTG	ATGCCAGTGAGCTTCCCGTTCAG
	*Has1*	GTGCGAGTGTTGGATGAAGACC	CCACATTGAAGGCTACCCAGTATC
	*Has2*	GCCATTTTCCGAATCCAAACAGAC	CCTGCCACACTTATTGATGAGAACC
	*Has3*	GCTTCAGTCCAGAAACCAAAGTAGG	CCTCGTTCCTCAAGAGAAACAAGG
	*Col1a1*	CTCGAGGTGGACACCACCCT	CAGCTGGATGGCCACATCGG
	*Col1a2*	AAGGGTGCTACTGGACTCCC	TTGTTACCGGATTCTCCTTTGG
	*Mmp‐1*	CCCCATACTGATGGACGTGG	TCCTCTCTTGAAAGGAGATGCC
	*Sod*	GGGAAGCATGGCGATGAAAG	CCCCATACTGATGGACGTGG
	*Cat*	TTTTGCCTACCCGGACACTC	GGGGTAATAGTTGGGGGCAC
	*Gpx2*	AATGTGGCGTCACTCTGAGG	GGGAAGCCGAGAACTACCAG

### 2.8 Statistical Analysis

All experiments were performed at least in triplicate, and the results presented in the figures are expressed as mean ± standard deviation. Statistical significance was determined using Student's *t*‐test analysis or one‐way ANOVA followed by Dunnett's multiple comparison test. Results were considered significant at *p* < 0.05.

## 3 Results

### 3.1 Safety Evaluation of SEP Administration

**FIGURE 1:**
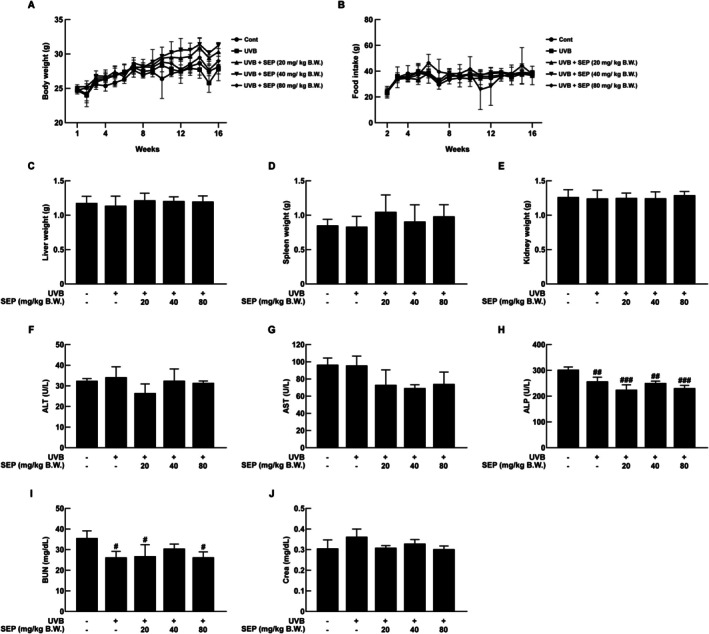
SKH‐1 hairless mice administered orally with SEP showed no toxicity. (A, B) Body weight and food intake were measured once per week. (C–E) Liver, spleen, and kidney tissues weight measurements for each group. (F–J) Blood analysis of mice, measuring AST, ALT, ALP, BUN, and CREA levels (^#^ *p* < 0.05, ^##^ *p* < 0.01, ^###^ *p* < 0.001 compared with untreated control group; o mark indicates no significant difference compared with the UVB‐only group).

The safety of oral administration of SEP was evaluated in SKH‐1 mice. No significant differences were observed in body weight changes and food intake among all groups in 16 weeks (Figure 1A,B). Similarly, there were no differences in the weights of the liver and kidneys (Figure 1C,E). The spleen weight in the 20 mg/kg body weight (B.W.) group was higher, but the difference was not statistically significant (Figure 1D). To check if SEP could be harmful, we measured the amounts of alanine aminotransferase (ALT), aspartate aminotransferase (AST), alkaline phosphatase (ALP), blood urea nitrogen (BUN), and creatinine in the serum. As shown in Figure 1F,G,J, there were no significant differences in ALT, AST, and ALP levels between the untreated control group and the other experimental groups. However, as shown in Figure 1H,I, ALP and BUN levels tended to decrease in the SEP‐treated groups. These results suggest that oral administration of SEP does not show toxicity in vivo. In addition, statistical comparisons between the UVB‐only and UVB + SEP groups revealed no significant differences (NS).

### 3.2 SEP Alleviates UVB‐Induced Moisture Loss and Wrinkle Formation on Skin

**FIGURE 2:**
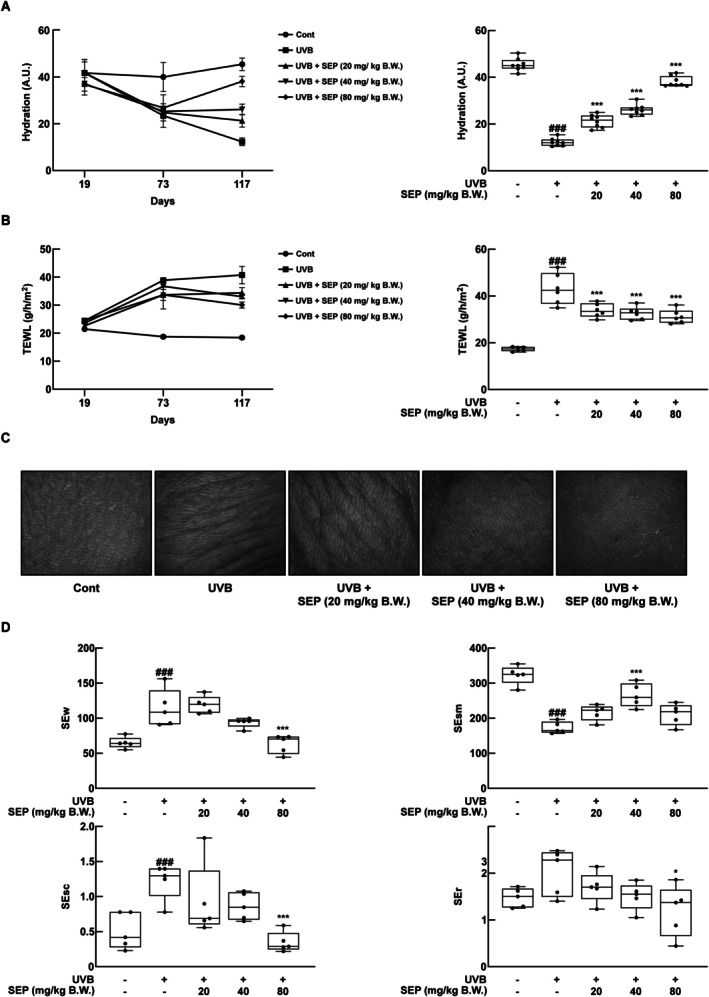
SEP inhibits UVB‐induced wrinkle formation and dehydration. (A) Skin moisture content was measured at the early, middle, and final stages of the experiment. (B) Trans epidermal water loss (TEWL) was measured at the early, middle, and final stages of the experiment. (C) Wrinkle images of the dorsal skin were captured at the end of the experiment. (D) Four wrinkle parameters (Sew, SEsm, SEsc, SEr) shown in the images in (C) were analyzed. (^###^ *p* < 0.001 compared with untreated control group; *p < 0.05, ***p < 0.001 compared with UVB‐only group).

Skin is the tissue most exposed to UVB radiation. UVB causes moisture depletion and disrupts the skin's permeability barrier, leading to increased trans epidermal water loss (TEWL) (Haratake et al. 1997). To evaluate the protective effect of SEP on UVB‐induced skin moisture depletion, changes in skin hydration content were measured in SKH‐1 hairless mice. It was observed that the skin hydration in the group exposed to UVB decreased significantly compared to the group that wasn't exposed, and SEP helped to gradually bring back the moisture lost due to UVB (Figure 2A). Additionally, TEWL, which indicates the evaporation of moisture from the skin, was also found to be increased by UVB exposure, and SEP treatment significantly reduced this moisture loss (Figure 2B).

UVB irradiation leads to the degradation of collagen and promotes hyperkeratinization, which induces wrinkle formation and becomes a major cause of overall skin health damage (Imokawa and Ishida 2015). In tests on animals, exposing mice to UVB caused wrinkles and their skin to be thicker on their backs (Figure 2C). Measurements of wrinkles (SEw), skin smoothness (SEsm), scaliness (SEsc), and skin roughness (SEr) using the Visioscan VC 20plus revealed that, except for SEsm, all parameters in the UVB and SEP co‐treatment group were significantly improved by SEP treatment, restoring them to levels like those of the unexposed group (Figure 2D). These results demonstrate that SEP inhibits UVB‐induced wrinkle formation and improves overall skin texture.

### 3.3 SEP Reduces UVB‐Induced Collagen Loss and Epidermal Thickening

**FIGURE 3:**
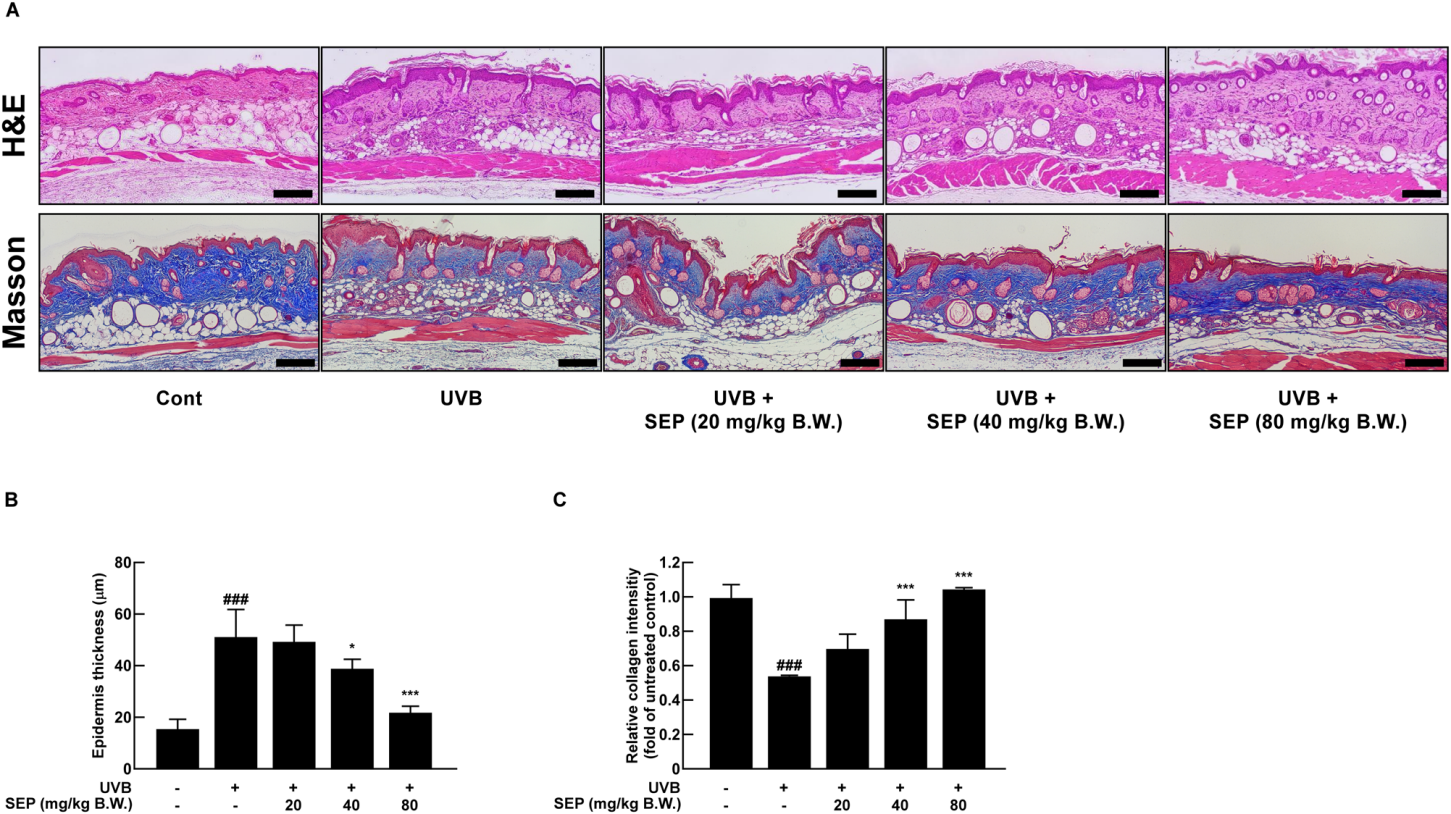
SEP prevents UVB‐induced collagen loss and epidermal thickening in dorsal skin tissue in SKH‐1 mice. (A) H&E staining and Masson's trichrome staining were performed to evaluate epidermal thickness and collagen content, respectively. Scale bar = 200 μm. (B) Epidermal thickness was quantified based on the H&E staining results. (C) Collagen content was quantified based on Masson's trichrome staining results. (^###^ *p* < 0.001 compared with untreated control group; *p < 0.05, ***p < 0.001 compared with UVB‐only group).

UVB exposure makes the outer epidermis thicker, causes an increase in the production of skin cells, and boosts the activity of enzymes like MMP‐1 that break down collagen, a key protein for maintaining skin firmness and elasticity, leading to faster skin aging (Hachiya et al. 2009). To assess how this affects epidermal thickness and collagen levels, H&E staining was used to measure the thickness of the epidermis, while Masson's trichrome staining was used to measure the amount of collagen present (Figure 3A). Image J software analysis revealed that UVB exposure significantly increased epidermal thickness, while SEP treatment dose‐dependently reduced this thickening. Notably, the 80 mg/kg B.W. group showed a complete recovery to levels comparable to the non‐irradiated group (Figure 3C). In Figure 3A, the blue‐stained area in the dermal layer represents collagen content. Image J quantification showed that collagen levels in the UVB‐only group were significantly reduced compared to the non‐exposed group, but SEP treatment dose‐dependently restored collagen content. These results suggest that SEP helps reduce the thickening of the skin and loss of collagen caused by UVB, which helps keep the skin healthy.

### 3.4 SEP Ameliorates UVB‐Induced Skin Damage

**FIGURE 4:**
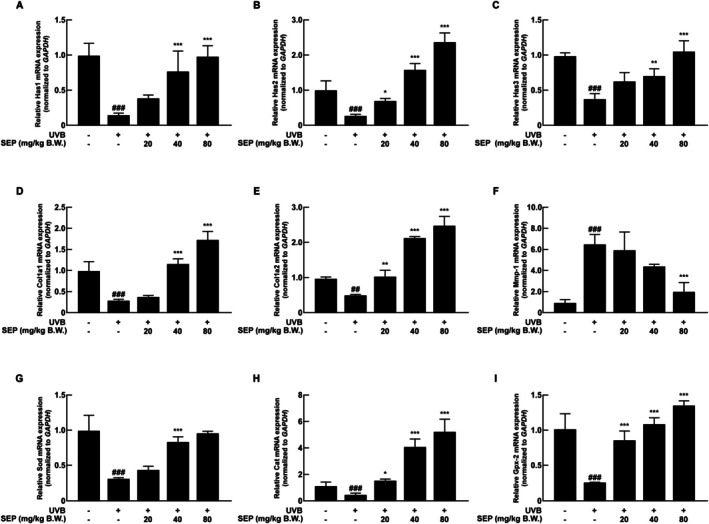
The effect of SEP on the mRNA expression levels of skin health‐related markers was assessed. (A–C) mRNA expression levels of *Has1*, *Has2*, and *Has3*, which are markers of skin hydration, were analyzed. (D, E) mRNA expression levels of *Col1a1* and *Col1a2*, markers of skin wrinkles, were examined. (F) The mRNA expression level of *Mmp‐1* was measured. (G) The mRNA expression level of the antioxidant marker *Sod* was assessed. (H) The mRNA expression level of *Cat* was analyzed. (I) The mRNA expression level of *Gpx2* was examined. (^##^ *p* < 0.01, ^###^ *p* < 0.001 compared with the untreated control group; n*p < 0.05, **p < 0.01, ***p < 0.001 compared with the UVB‐only group).

Hyaluronan synthase (HAS) is an enzyme that makes hyaluronic acid (HA), a type of glycosaminoglycan that is important for keeping the skin hydrated, especially in conditions like xerosis (Zheng et al. 2023). As shown in Figure 4A, oral administration of SEP helped to gradually bring back the lower levels of Has1 mRNA caused by UVB exposure. Similarly, SEP treatment significantly restored the expression levels of Has2 and Has3 mRNA (Figure 4B,C).

Type I collagen is a key protein that helps form structures in the body, mainly produced by the COL1A1 and COL1A2 genes (Lu et al. 2019). Matrix metalloproteinase‐1 (MMP1) degrades various extracellular matrix (ECM) components and contributes to skin aging (Pittayapruek et al. 2016). As shown in Figure 4D,E, oral administration of SEP greatly raised the levels of COL1A1 and COL1A2 mRNA, which had dropped due to UVB exposure. Also, the increase in MMP1 mRNA levels caused by UVB exposure was reduced from six times to about two times after treatment with SEP, suggesting that SEP may help prevent the breakdown of collagen (Figure 4F).

Superoxide dismutase (SOD) plays a protective role against oxidative stress‐induced skin dysfunction (Shariev et al. 2021; Zhu et al. 2024). As shown in Figure 4G, treating SEP restored the lower levels of SOD mRNA caused by UVB, making it like the levels seen in the control group. An increase in SOD may indirectly suggest the suppression of ROS‐mediated effects, indicating its potential role in modulating oxidative stress and inflammation. In addition to SOD, SEP dose‐dependently increased the mRNA levels of catalase (Cat) and glutathione peroxidase 2 (Gpx2) which were significantly reduced by UVB irradiation (Figure 4H,I). These enzymes are critical for detoxifying hydrogen peroxide and lipid hydroperoxides, thereby complementing the antioxidant defense provided by SOD (Brigelius‐Flohe and Maiorino 2013; Chelikani et al. 2004). These findings suggest that SEP has strong protective effects against UVB‐induced skin deterioration by influencing several processes related to skin moisture, collagen production, and resistance to oxidative stress.

## 4 Discussion

UVB exposure is a major factor in skin aging and damage (Piernick et al. 2019). While topical and external strategies have been widely explored, oral interventions remain limited. This study provides novel evidence that oral administration of Edible Snail Extract Powder confers significant protective effects against UVB‐induced skin damage, including improved hydration and reduced wrinkle formation. Additionally, although the present study was conducted with three dose levels (20, 40, and 80 mg/kg B.W.), future research should establish a dose–response curve to identify the optimal dose that exerts beneficial effects on skin health.

One of the key findings of this study is that SEP restored the expression of HAS that was downregulated due to UVB exposure. Hyaluronic acid plays a critical role in maintaining skin moisture (Everett and Sommers 2013). In our study, oral administration of SEP significantly increased the mRNA expression levels of *HAS1*, *HAS2*, and *HAS3*. These findings suggest that SEP promotes hyaluronic acid synthesis in the dermis, thereby mitigating skin dehydration caused by UVB. This molecular effect was consistent with the observed improvements in skin moisture content and reductions in TEWL.

SEP also contributed to the improvement of skin structure by modulating the expression of collagen metabolism‐related genes. The increased expression of *Col1a1* and *Col1a2*, along with the suppression of the collagen‐degrading enzyme Mmp‐1, indicates that SEP promotes collagen synthesis while simultaneously inhibiting its breakdown, thereby helping to preserve dermal structure. These molecular changes were further supported by histological results, which showed that SEP alleviated UVB‐induced epidermal thickening and restored collagen content.

Moreover, SEP restored the expression of the antioxidant enzyme *SOD*, suggesting its role in protecting the skin through suppression of oxidative stress. In addition, SEP dose‐dependently increased the expression of *Cat* and *Gpx2*, two key enzymes responsible for detoxifying hydrogen peroxide and lipid hydroperoxides (Brigelius‐Flohe and Maiorino 2013; Chelikani et al. 2004), thereby complementing the antioxidant defense provided by *SOD*. Although our study focused on mRNA expression, previous studies reported that snail mucin suppressed UVB‐induced MMP‐1 expression and reduced c‐Jun activation at the protein level, supporting the translational relevance of our findings (Kim et al. 2022). Given that excessive ROS generation activates MAPK/AP‐1 signaling (Glady et al. 2018), the reinforcement of multiple antioxidant enzymes by SEP likely contributes to limiting oxidative stress and downstream inflammatory responses.

Although further mechanistic studies, including cellular experiments and gut microbiome analysis, are needed, our findings demonstrate that oral administration of SEP effectively regulates key biomarkers related to skin hydration, collagen metabolism, and oxidative defense. These results suggest that SEP is a promising functional food ingredient for skin protection and may serve as a valuable candidate for development as a nutricosmetic agent. Future studies investigating the molecular interactions within the gut–skin axis and the effects on wound healing would provide a deeper understanding of the impact of SEP on skin health.

## Author Contributions

**Jamyeong Koo:** formal analysis (equal), writing – original draft (equal). **Sungmin Cho:** formal analysis (equal), investigation (equal). **Wonchul Lim:** supervision (equal), writing – review and editing (equal). **Tae‐Gyu Lim:** project administration (equal), supervision (equal), writing – review and editing (equal).

## Conflicts of Interest

The authors declare no conflicts of interest.

## Data Availability

The data that support the findings of this study are available on request from the corresponding author.
